# Factors Affecting Splicing Strength of Yeast Genes

**DOI:** 10.1155/2011/212146

**Published:** 2011-11-20

**Authors:** Pinchao Ma, Xuhua Xia

**Affiliations:** ^1^Department of Biology, University of Ottawa, 30 Marie Curie, P.O. Box 450, Station A, Ottawa, ON, Canada K1N 6N5; ^2^Ottawa Institute of Systems Biology, University of Ottawa, Ottawa, ON, Canada K1H 8M5

## Abstract

Accurate and efficient splicing is of crucial importance for highly-transcribed intron-containing genes (ICGs) in rapidly replicating unicellular eukaryotes such as the budding yeast *Saccharomyces cerevisiae*. We characterize the 5′ and 3′ splice sites (ss) by position weight matrix scores (PWMSs), which is the highest for the consensus sequence and the lowest for splice sites differing most from the consensus sequence and used PWMS as a proxy for splicing strength. *HAC1*, which is known to be spliced by a nonspliceosomal mechanism, has the most negative PWMS for both its 5′ ss and 3′ ss. Several genes under strong splicing regulation and requiring additional splicing factors for their splicing also have small or negative PWMS values. Splicing strength is higher for highly transcribed ICGs than for lowly transcribed ICGs and higher for transcripts that bind strongly to spliceosomes than those that bind weakly. The 3′ splice site features a prominent poly-U tract before the 3′AG. Our results suggest the potential of using PWMS as a screening tool for ICGs that are either spliced by a nonspliceosome mechanism or under strong splicing regulation in yeast and other fungal species.

## 1. Introduction

Introns in eukaryotic genes are spliced out mainly by the spliceosome [1] through a multitude of RNA-RNA, RNA-protein, and protein-protein interactions involving the 5′ splice site (ss), the 3′ ss, and the branch point sequence [2–10]. The yeast, *S. cerevisiae, *appears to have only U2-type introns [11, 12], with the consensus sequences of 5′ ss and 3′ ss being 5′-∣GUAUGU and YAG∣-3′, respectively [11, 13, 14]. 5′ ss is strongly constrained by base pairing with U1 and U6 snRNAs [15–17], leading to an overwhelming majority of 5′ ss having the consensus of GUAUGU in the yeast. In multicellular eukaryotes and in fission yeast, 3′ ss is strongly constrained by U2AF^35^ proteins [18]. However, no *U2AF35* homologue has been found in the budding yeast [19, 20], although 3′ ss is known to be partially constrained by the PRP8p protein [21].

A gene whose protein needs to be mass produced would need not only to have a high transcription rate, but also to possess features allowing it to be spliced efficiently and accurately. Thus, splicing is a major component of the quality control process in mRNA production in eukaryotes [22]. Highly expressed genes should evolve to have efficient 5′ ss and 3′ ss to avoid aberrant splicing which is not only wasteful but can also produce wrong proteins that perturb the normal cellular processes. In contrast, the selection for high splicing strength should be relatively weak in lowly expressed genes whose ss may drift to low splice efficiency through mutation. This has two implications concerning splicing strength. First, splicing strength of highly transcribed genes should, on average, be higher than that of lowly transcribed genes. Second, the variance of splicing strength should be larger for lowly transcribed genes (whose splicing strength could be high but may also drift to low values through mutation) than that for highly transcribed genes (whose splicing strength should all be high). This paper presents a first systematic analysis of the relationship between splicing strength and the level of gene expression.

A comprehensive assessment of the relationship between intron splicing strength and gene expression requires accurate characterization of introns and reliable large-scale measurement of gene expression. The yeast (*S. cerevisiae*) is the first species with accurate characterization of its introns and gene expression at mRNA and protein levels. Two powerful methods have recently been developed to characterize yeast introns. The first is to use high-density yeast tiling arrays in conjunction with a yeast mutant deficient for degradation of processed intron lariats [23]. The accumulation of lariats in the RNA pool is detected by the high-density tiling array which allows not only intron validation but also detection of new introns. The second approach involves designing microarray probes specific for exon-intron junctions and exon-exon junctions to quantitatively characterize unspliced and spliced mRNA [24–26]. Furthermore, *S. cerevisiae* is one of the few species with large-scale genome-wide characterization of both mRNA transcripts [27, 28] and protein abundance [29] or protein synthesis rate [30]. While transcripts and proteins have now been characterized for other species as well, there is no species in which introns have been characterized as accurately and thoroughly as the yeast. In this study, we use the yeast data to investigate the relationship between gene expression and splicing strength. 

Other than the availability of high-quality molecular data, there are additional advantages in using *S. cerevisiae *for such a study. First, the yeast cells need to replicate rapidly and natural selection should act strongly against highly expressed yeast genes with poorly spliced introns. Second, the yeast genome has few introns, and most of them have been correctly annotated [23, 24, 31, 32]. Third, the splicing mechanism in the yeast is relatively simple compared to higher eukaryotes [24], with key spliceosome proteins better characterized than any other organisms [33]. Fourth, except for a few genes [31, 34], alternative splicing observed in multicellular eukaryotes is rare in the yeast [35]. The splicing mechanism in *S. cerevisiae *appears to be simple even among fungal species, for example, its genome does not have homologs of the U2AF^35^ spliceosomal protein which is present in other fungal species such as the fission yeast (*Schizosaccharomyces pombe*) as well as multicellular eukaryotes with sequenced genomes [19, 20]. *S. cerevisiae *also lacks the serine-arginine proteins serving as essential splicing factors in metazoans [36].

It is difficult to measure splicing strength directly, and previous publications have used the position weight matrix (PWM, [37, 38] for detailed numerical illustration) derived from the ss and the resulting PWM score (PWMS) as a proxy for splicing strength [39, 40]. If an overwhelming majority of introns are spliced by the spliceosome mechanism, if there is an optimal state of the ss strongly preferred by the spliceosome mechanism (i.e., introns with ss in their optimal state are most efficiently spliced), and if there is strong selection pressure to maintain such an optimal state for most of the genes (i.e., if mutations leading to deviation from such an optimal state are deleterious), then we should expect that most ss should converge towards the optimal state, that the ss with the optimal state will have the highest PWMS, and that those deviating from the optimal state should have low PWMS. In short, PWMS may be used as a proxy for splicing strength by the spliceosome mechanism if the three conditions are satisfied. Hereafter, splicing strength refers specifically to splicing strength by the spliceosome mechanism.

## 2. Materials and Methods

### 2.1. 5′ and 3′ Splice Sites

The genomic sequences of all 16 chromosomes of *Saccharomyces cerevisiae* were retrieved from ftp://ftp.ncbi.nlm.nih.gov/genomes/Fungi/Saccharomyces_cerevisiae_uid128/ (assembly date: 14-JUL-2011). There are 279 annotated introns breaking the coding region in 270 genes, with 261 genes each containing a single intron and nine genes (SUS1, VMA9, HMRA1, DYN2, YOS1, RPL7A, AML1, TAD3, and RPL7B) each containing two introns. Some introns from paralogous genes are identical. Genes YBL111C, YHR218W, YLL067C, YLL066C, and YML133C are paralogous and contain the same intron, so are the genes YIL177C and YJL225C and the genes YRF1-3, YRF1-6, and YRF1-7. This creates two problems. The first involves the lack of data independence in statistical analysis. The second involves the quantification of mRNA and protein production. Take genes YIL177C and YJL225C, for example. It is difficult to know if the mRNA and protein abundance is contributed by only one of the two genes or by both. However, paralogues are few among yeast ICGs, and excluding these genes from analysis does not alter the conclusions reached in this paper.

There are 24 genes with introns in the 5′-UTR (Table 1). We originally thought that they might have weaker ss than those located within coding sequences because the failure to splice such introns seems to have little functional consequence as long as translation machinery can find the proper translation initiation site. However, there is no detectable difference between the two. Excluding or including these 24 yeast ICGs does not alter the conclusion in the paper.

For each intron, we originally extracted 10 bases from the exon side and 12 bases from the intron side by using DAMBE [41, 42]. This 10 + 12 configuration excluded some ss because the first exon in some yeast genes is shorter than 10 bases (note that the term “first exon” refers to the coding part of the first exon in this paper). For example, the first exon of the two-exon *MUD1* gene is only eight bases long. Because our extraction requires 10 bases on the exon side, 5′ ss of such genes would be missed. The most extreme cases of this are the *RPL20A *and *RPL20B *genes which have a single nucleotide as their first exon, that is, in the configuration of 5′-A∣intron∣TG-3′. With the requirement of 10 + 12 configuration, the total number of 5′ ss is only 223 in the yeast genome. As a preliminary analysis revealed that only five sites on the exon side of 5′ ss showed significant sequence conservation, we defined our 5′ ss to consist of 5 nucleotides on the exon site and 12 nucleotides on the intron side (referred to hereafter as the 5 + 12 configuration). Similarly, a 3′ ss consists of 12 nucleotides on the intron side and 5 nucleotides on the exon side. This results in 275 5′ ss and 301 3′ ss that have the 5 + 12 configuration, including the 24 introns in 5′UTR.

Some researchers (e.g., [39, 43]) have taken 5′ ss to span from the last 3 nucleotides of the exon to the first 6 or 7 nucleotides of the intron. 5′ ss defined in this way may produce spurious site patterns in the yeast. For example, as shown in Table 2 which lists genes with their 5′ ss excluded due to too short upstream exon, 20 *S. cerevisiae *genes have first exons with exactly three nucleotides (i.e., containing only the initiation codon). Defining 5′ ss with three nucleotides in the exon side will substantially increase the representation of A, U, and G at the three nucleotide sites (i.e., the −3, −2, and −1 sites) in 5′ ss (where the first nucleotide of the intron is labeled 1).

Some yeast introns might have been annotated incorrectly. The annotated intron in the *YJR112W-A* gene is the shortest intron in yeast (49 bp) and does not end with AG. It is possible that the intron is in fact longer with the real 3′ ss further downstream. According to SGD annotation [44], *YJR112W-A* is described as “putative protein of unknown function, identified based on homology to *Ashbya gossypii.*” So, we excluded its 3′ ss from our analysis. This reduces 303 3′ ss to 302.

### 2.2. Characterizing the Efficiency of Splicing Sites (ss) by Position Weight Matrix (PMW) and Sequence Logos

The consensus 5′ ss on the intron side in the yeast is GUAUGU. Thus, a simple approach to characterize 5′ ss splicing strength would be to give 5′ ss a high splicing strength value if it is similar to the consensus but a low value if it is entirely different from the consensus. A more formal approach is to characterize the ss by a position weight matrix (PWM, [37, 38] for detailed numerical illustration) and use the PWM score (PWMS) for each ss as its index of splicing strength [39, 40]. We used DAMBE [41, 42] to compute PWMS. 

The nucleotide frequencies of entire transcripts (i.e., including both exons and introns) were used as background frequencies for computing PWM, with A = 0.3279, C = 0.1915, G = 0.2043, and U = 0.2763. Because some site-specific frequencies are 0, a pseudocount with *α* = 0.0001 is added to all frequencies to avoid taking Log_2_ of 0 [38, pages 83–92]. An alternative is to specify the nucleotide frequencies of all exons as the background frequencies for the exon part of the ss and nucleotide frequencies of all introns as the background frequencies for the intron part of the ss. However, results thus obtained are similar to those using the first approach. We have also obtained results by using nucleotide frequencies of the extracted ss as background frequencies. The results are again similar.

Several studies [43, 45] assumed equal background frequencies in characterizing ss with PWM. This is not a good approach because it confounds the site-specific nucleotide bias at the ss with the genomic nucleotide bias. For example, the yeast genome is AT rich, and sequence segments assembled randomly from an AT-rich nucleotide pool will also be AT rich. Such random segments, when characterized by PWM with equal background frequencies, will appear informative and lead to false discovery of site patterns.

Another commonly used method for graphically displaying site-specific nucleotide patterns is the sequence logo which has been used to characterize intron ss [19]. The original method [46] does not take background nucleotide bias into consideration, and the resulting sequence logo is equivalent to a PWM assuming equal nucleotide frequencies. For example, AT-biased background frequencies in the yeast imply that the sequence logo will display A and T more prominently than C and G even when the sequences of interest contain no site-specific information. However, this problem has been eliminated by a recent improvement [47] which allows one to specify background (prior) frequencies just as in PWM. The sequence logographs in this paper are generated from the RNA Structure Logo website at http://www.cbs.dtu.dk/~gorodkin/appl/slogo.html.

### 2.3. Gene Expression

We used three measures of gene expression. The first is codon adaptation index [48] with its improved implementation in DAMBE [49], computed with the reference set of highly expressed yeast genes whose codon usage table is compiled in the Eysc_h.cut file distributed with EMBOSS [50]. The coding sequences (CDSs) for computing CAI were extracted by using DAMBE. CAI is intended to measure the efficiency of translation elongation but is highly and positively correlated with gene expression at the protein and mRNA level [51–53]. The advantage of using CAI is that it can be computed for all coding sequences, whereas empirical quantification of gene expression may be limited to relatively highly expressed genes which will not give us a whole picture of the relationship between splicing strength and gene expression.

The second measure of gene expression is the relative mRNA abundance of yeast genes from two previous studies that characterizes genome-wide RNA abundance in yeast [27, 28]. The microarray data [27] were downloaded from http://web.wi.mit.edu/young/pub/data/orf_transcriptome.txt. The data set includes mRNA levels for 5460 yeast genes. The absolute quantification data [28] is downloaded from the online supplementary material. Only the average expression in the YPD medium for 4817 genes was analyzed in this paper. 

The third measure of gene expression is the protein production of yeast genes characterized in two previous studies. The protein abundance data [29] were downloaded from http://www.nature.com/nature/journal/v425/n6959/extref/nature02046-s2.xls">nature02046-s2.xls. The predicted protein synthesis rate in two experimental conditions (mating pheromone treatment and control) was reliably measured for 3916 genes (Supplemental Table II in [30]), and we used the average of the two experiments. 

In the mRNA and protein characterization, YAR044W is synonymous to YAR042W in the GenBank file, so is YDR474C to YDR475C, YJL018W to YJL019W, YJL021C to YJL020C, YPR090W to YPR089W, and YFR024C to YFR024C-A. Some genes (YEL068C, YER084W, YHR173C, YIL054W, YJR146W, YLR358C, YNL140C, YNL143C, YNL184C, and YOR105W) were annotated in SGD as “dubious open reading frame unlikely to encode a protein”, and are not annotated at all in the *S. cerevisiae *genome in NCBI. However, they were found to be expressed at both mRNA [27] and protein levels [29] and are therefore included in our analysis. YFL006W and YFL007W have been merged into YFL007W, YJL017W and YJL016W into YJL016W, and YOR087W and YOR088W into YOR087W in the most recent yeast genome annotation. 

Two compiled data files are attached as supplementary materials. One (PWM-All.xls) includes all introns, mRNA abundance from the GATC-PCR method [28], and protein synthesis rate based on ribosomal loading and mRNA [30]. The other (PWM-No5UTRintrno.xls) excludes 5′ UTR introns and includes mRNA abundance from microarray [27] and protein abundance data [29].

## 3. Results and Discussion

### 3.1. Position Weight Matrix (PWM) and Its Statistical Significance

**Figure 1:**
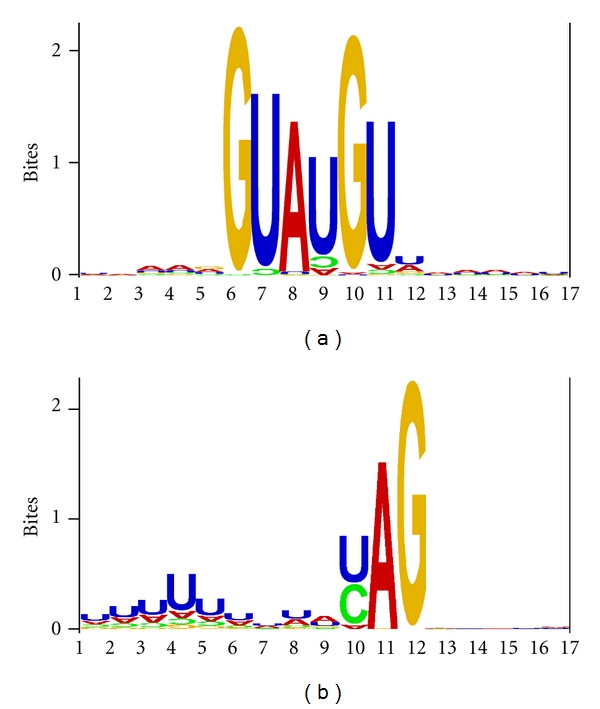
Sequence logos of 5′ ss (a) and 3′ ss (b), produced with the background frequencies specified as A = 0.3279, C = 0.1915, G = 0.2043, and U = 0.2763. The nucleotides whose frequencies are lower than expected are plotted upside down. The vertical bar is the information index computed as −[∑*P*_*i*_log⁡⁡_2_(*P*_*i*_)], where *P*_*i*_ is the frequency of nucleotide *i* ( = A, C, G  or  U) at each site.

Consistent with previous experimental studies on *S. cerevisiae*, the position weight matrices (Tables 3 and 4) and the sequence logos (Figure 1) not only confirmed but also expanded the consensus sequence of yeast splice sites, with UAAAG∣GUAUGUUUAAUU as the strongest 5′ ss and UUUUUUUUAYAG∣GCUUC as the strongest 3′ ss. Whether a PWM contains significant site-specific information can be tested by using the *F* statistic [37] defined as

(1) $$F=\overset{4}{\underset{i=1}{\sum }}\;\overset{L}{\underset{j=1}{\sum }}p_{ij}\ln\frac{p_{ij}}{p_{i}},$$

where *L* is the sequence length (motif width equal to 17 in our study), *i* = 1, 2, 3, and 4 corresponding to A, C, G, and U, *p*_*i*_ is the background nucleotide frequency for nucleotide *i*, and *p*_*ij*_ is the frequency of nucleotide *i* at position *j*( = 1,2,…, 17). A straightforward method for evaluating the significance of PWM is by resampling. With the tetranomial distribution defined by (*p*_A_+*p*_C_+*p*_G_+*p*_T_)^*L*^, we can obtain a new set of sequences (e.g., 246 sequences of 17 nt each) and compute *F*. This is repeated for, say, 5000 times to obtain 5000 *F* values. The 95th or 99th percentile of the *F* values can be taken as critical *F* values at 0.05 and 0.01 significance levels, respectively. An observed *F* for the PWM is significant if it is greater than the critical *F*. Based on this criterion, the PWM from the 275 5′ ss and that from the 301 3′ ss are both highly significant (*P* < 0.0001). It is also highly significant (*P* < 0.0001) when the 24 introns in 5′ UTR are excluded.

Given the significant PWM for 5′ ss and 3′ ss, we want to know which individual nucleotide sites (out of 17 in total) contribute to the significance. All 17 nucleotide sites of 5′ ss and 16 nucleotide sites of 3′ ss are significant at 0.05 level when experimentwise error rate is not controlled for (Tables 3 and 4). One popular statistical method for controlling experimentwise error rate is the method of false discovery rate (FDR) [54, 55]. The classical FDR approach [54], commonly referred to as the Benjamini-Hochberg procedure or simply the BH procedure, sorts *p* values in ascending order and computes *p*_critical.BH.*i*_ (where the subscript BH stands for the BH procedure) for the *i*th  *p* value as

(2) $$p_{\text{critical}.\text{BH}.i}=\frac{q\cdot i}{N},$$

where *q* is FDR (e.g., 0.05), *i* is the rank of the *p* value in the sorted array of *p* values, and *N* is the number of tests (i.e., the number of *p* values, 17 in our case). If *k* is the largest *i* satisfying the condition of *p*_*i*_ ≤ *p*_critical.BH.*i*_, then we reject hypotheses from *H*_1_ to *H*_*k*_. In our case, all the 17 nucleotide sites are statistically significant based on *p*_critical.BH.*i*_ (Table 5).

The FDR procedure above assumes that the test statistics are independent or positively dependent and a more conservative FDR procedure has been developed that relaxes the assumption [55]. This method, commonly referred to as the Benjamini-Yekutieli or simply the BY procedure, computes *p*_critical.BY.*i*_ for the *i*th hypothesis as

(3) $$p_{\text{critical}.\text{BY}.i}=\frac{q\cdot i}{N\sum_{i=1}^{N}1/i}=\frac{p_{\text{critical}.\text{BH}.i}}{\sum_{i=1}^{N}1/i}.$$

With *N* = 17 in our case, ∑1/*k* = 3.439552523. Based on *p*_critical.BY.*i*_, nucleotide sites −5 and −4 in 5′ ss are not statistically significant (Table 5).

All 17 nucleotide sites of 3′ ss are also significant at the 0.05 level based on the criterion of *p*_critical.BH.*i*_. However, with the more conservative criterion of *p*_critical.BY.*i*_, the five nucleotide sites on the exon side are not significant.

There is no significant difference in 5′ and 3′ ss PWMS between the 24 introns in 5′ UTR and those in the coding regions (*P* = 0.1606 for 5′ ss PWMS and *P* = 0.3182 for 3′ ss PWMS). The two sets are pooled in the rest of the analysis.

### 3.2. Gene Expression and Splicing Strength

**Figure 2:**
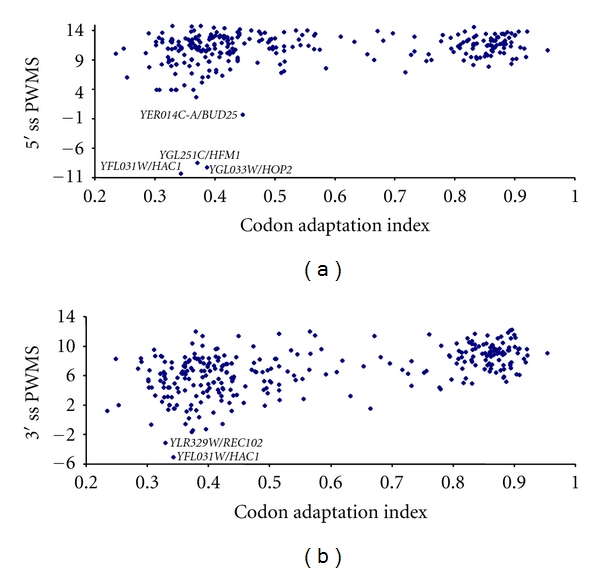
Relationship between splicing strength measured by position weight matrix score (PWMS) at 5′ (a) and 3′ (b) splice sites (5′ ss and 3′ ss) and gene expression measured by codon adaptation index.

**Figure 3:**
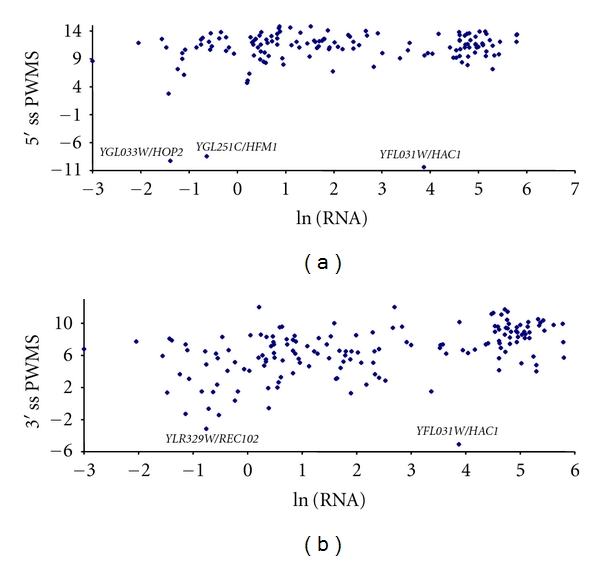
Relationship between splicing strength measured by position weight matrix score (PWMS) at 5′ (a) and 3′ (b) splice sites (5′ ss and 3′ ss) and gene expression measured by mRNA abundance [28]. The mRNA abundance is log transformed. A similar pattern is observed when the mRNA abundance from Holstege et al. [27] is used.

**Figure 4:**
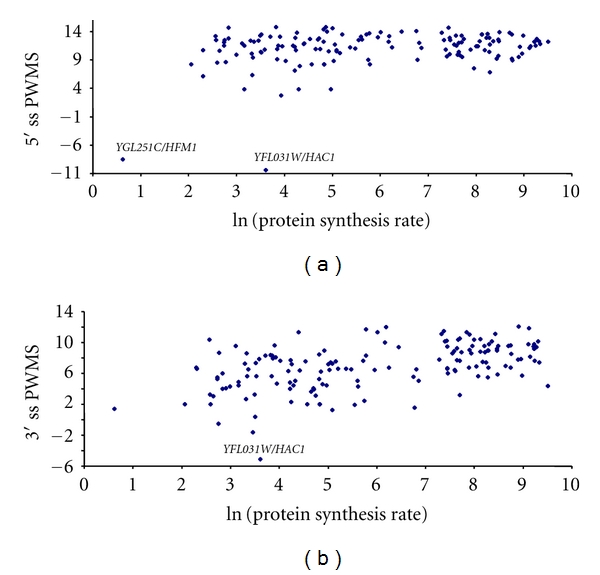
Relationship between splicing strength measured by position weight matrix score (PWMS) at 5′ (a) and 3′ (b) splice sites (5′ ss and 3′ ss) and gene expression measured by protein synthesis rate [30] which is log transformed. A similar pattern is observed when the protein synthesis rate is replaced by protein abundance from Ghaemmaghami et al. [29].

We have argued previously that lowly expressed genes will, on average, have introns with lower splicing strength (as measured by PWMS) but greater variance in PWMS than highly expressed genes. The splicing strength characterized by PWMS exhibited expected relationship with gene expression when the latter is measured by either CAI (Figure 2), mRNA abundance (Figure 3), or protein production (Figure 4). In addition, lowly expressed genes have greater variation in PWMS values than highly expressed genes. To statistically test the differences in mean and variance, we have ranked genes by gene expression, that is, ranked separately by CAI, mRNA abundance, or protein production. For each ranking, we designate 1/3 of the genes with the highest expression values (i.e., highest CAI, mRNA, or protein production, resp.) as the high-expression group and another 1/3 of the genes with the lowest expression values as the low-expression group and tested the differences in mean PWMS and the variance of PWMS between the two groups. As shown in Table 7, the two predictions are consistently supported, that is, (1) the highly expressed genes have significantly greater mean PWMS values than lowly expressed genes and (2) the highly expressed genes have significantly smaller variance in PWMS than the lowly expressed genes (Table 7). The *t*-tests used assume unequal variances between the two groups. The tests for differences in variance between the two groups are regular variance ratio *F*-test [56, pages 136–139].

The cluster of points in Figure 2 with CAI greater 0.8 and that in Figure 3 with ln(mRNA) greater than 3 are almost all ribosomal protein-coding genes which are highly transcribed [57] and have strong splicing sites (high PWMS). For these genes, mutations that weaken the splicing strength of their splicing sites are expected to be deleterious. Our result suggests that natural selection may be involved in maintaining high splicing strength in the splice sites of highly expressed genes.

### 3.3. Introns with the Poorest PWMSs for Their ss Are Spliced by Nonspliceosome Mechanisms or Require Additional Splicing Factors

5′ ss in three genes (*HAC1, HFM1, *and *HOP2*) have the most negative PWMSs (−10.3544, −9.2192, and −8.4717, resp.). Such PWMSs imply that 5′ ss of these genes have evolved to avoid being spliced by the spliceosomal mechanism because a random 17mer assembled from the nucleotide pool with the nucleotide frequencies of yeast protein-coding genes (A = 0.3279, C = 0.1915, G = 0.2043, and U = 0.2763) would have an expected PWMS of zero.

It is now known that the splicing of the pre-mRNA of these genes requires either a nonspliceosomal mechanism or additional protein factors for intron removal. *HAC1*, which plays a key role in the unfolded protein response (UPR) by binding to the UPR element [58–62], is one of the few yeast genes whose first exon is much longer than the second (661 bp and 56 bp, resp.), and its transcript is processed by an unconventional mechanism (i.e., nonspliceosomal splicing), with the intron cleaved by the protein kinase Ire1p, which possesses endonuclease activity and tRNA ligase [61, 63–65].

The *HFM1/MER3 *and *HOP2* are both meiosis-specific genes, with *HFM1 *coding for a meiosis-specific DNA helicase [66, 67] that participates in crossover control and unwinding of Holliday junctions [66–70] and *HOP2 *coding for a protein essential for forming meiotic synapsis between homologous chromosomes [71, 72]. The splicing of their transcripts is not constitutive but strictly regulated. The splicing of the *HFM1/MER3 *transcripts is regulated by the Mer1p and Bud13p proteins [73–75]. Unspliced *HOP2 *transcripts accumulate when the cell is not in meiosis [33]. The splicing of the *HOP2 *transcripts depends heavily on the nuclear exosome component Rrp6 protein, with the loss of *RRP6 *dramatically decreasing the splicing of *HOP2 *transcripts [33].

Other than the three genes above, the gene with the smallest 5′ ss PWMS is *BUD25* with its PWMS equal to 0.4267. Yeast spliceosome does not bind to *BUD25 *transcripts during transcription [33]. The *BUD25* gene is also implicated in chromosome segregation and meiosis [76]. Most yeast introns can be deleted with no effect, but deletion of *BUD25* intron causes defective growth [76], suggesting that splicing is important for its function and that its ss may be under additional constraints other than splicing strength. In other words, the ss of *BUD25 *may not be free to evolve towards high splicing strength.

3′ ss of several genes also have negative PWMSs. The intron of the *HAC1 *gene, which is spliced by a nonspliceosome mechanism [61, 63–65], has a 3′ ss with the smallest PWMS (−5.1038). The intron whose 3′ ss has the second smallest PWMS (−3.1252) belongs to *REC102* which is also a meiosis-specific gene, required for chromosome synapsis [77–79]. The splicing of its intron also makes use of a nonspliceosome mechanism [31]. Based on *in vitro* experiments, splicing of *REC102* message by yeast spliceosome is both inefficient [80] and inaccurate [80, 81], leading to many unspliced and wrongly spliced mRNAs. However, in a large-scale characterization of yeast total transcripts, only a single correctly spliced mRNA is found (file S03052-07_G10.seq in the online supplementary material in [82]). The amino acid sequence from the correctly spliced mRNA is highly conserved among different *Saccharomyces* species [80, 83–85]. Taken together, these results suggest that the *in vivo *correct splicing of *REC102 *pre-mRNA requires additional factors at the meiosis stage. These yeast ICGs whose intron splicing requires a nonspliceosome mechanism or additional splicing factors are poor in recruiting U1 snRPNs [33]. The result that such genes are strongly regulated and have low or negative PWMS indicates the potential of using bioinformatic methods to identify these strongly regulated genes.

### 3.4. A Prominent Poly-U Upstream of the AG Dinucleotide in 3′ ss

Efficiently spliced introns in the yeast are characterized by a poly-U tract upstream of the 3′AG (Table 4 and Figure 1). This trend is stronger when we exclude the yeast ICGs whose transcripts bind poorly to spliceosomes (result not shown). Such a poly-U tract can increase the efficiency of 3′ ss that has previously been demonstrated in *S. cerevisiae *[86], especially in introns with a long distance between the branch point site and 3′ ss [87]. A recent study of intron splicing of mammalian genes in YACs (yeast artificial chromosome) is consistent with the proposed importance of the poly-U tract upstream of 3′ ss in *S. cerevisiae *[88]. 

Previous compilations of yeast introns [11, 13] have missed the poly-U tract upstream of 3′ ss. Thus, the poly-U tract upstream of 3′ ss has not been included as a feature of *S. cerevisiae* intron in molecular biology textbooks (e.g., [14, page  428]). 

The poly-U tract upstream of the yeast 3′ ss is different from the polypyrimidine tract (where both U and C are overrepresented) that is often present upstream of 3′ ss in multicellular eukaryotes as well as in *Schizosaccharomyces pombe. *In *S. cerevisiae, *only U is overrepresented and C is underrepresented (shown backwards in the sequence logo for 3′ ss in Figure 1(b)). In multicellular eukaryotes and *S. pombe, *the polypyrimidine tract upstream of 3′ ss is important for splicing strength [89] and is recognized by the essential U2AF^65^ splicing factor [90]. However, while U2AF^65^ is highly conserved from *S. pombe *to multicellular eukaryotes, the U2AF^65^ homologue in the budding yeast, MUD2p, is highly diverged and not essential for survival [20]. This may have contributed to the evolutionary origin of the poly-U tract in the budding yeast.

The presence of poly-U implies that the recognition of 3′ ss may involve more than simple scanning for the first AG after the branchpoint site. In fact, it has previously been shown that a proximal PyAG without poly-U is often skipped if a more distal PyAG occurs with a poly-U [86].

### 3.5. Yeast ICGs Weakly Bound to U1 snRNPs Have Smaller PWMS Than Those Bound Strongly

A recent study [33] documented 50 yeast ICGs whose mRNA failed to recruit U1 snRNPs to the site of transcription in detectable amount. We tested the possibility that these genes may have weak ss by comparing PWMS between these 50 genes and other yeast ICGs. Three of these 50 genes (YOR074C, YOR221C, and YLR312W-A) actually do not have introns and should not be included as yeast ICGs. In addition, YOR318C is a dubious gene with no *in vivo* evidence, that it is, putative intron is spliced. The remaining 46 genes have 48 introns, with YCL005W-A and YCR097W each having two introns. The mean PWMS is 8.8138 for the 5′ ss of these 48 introns and 11.1978 for the rest of introns. The difference is highly significant based on a two-sample *t*-test (DF = 273, *t* = −4.6346, *P* < 0.0001, two-tailed test, Table 6). The same pattern is seen for 3′ ss (Table 6). Thus, Yeast ICGs weakly bound to U1 snRNPs during transcription have weaker 5′ and 3′ ss than those bound strongly to U1 snRNPs.

It is not clear why Yeast ICGs weakly bound to U1 snRNPs during transcription should have weak 3′ ss because 3′ ss is not expected to be involved in recruiting U1 snRNPs during transcription. One possible explanation is that a weak 5′ ss that does not recruit U1 efficiently tends to be associated with a weak 3′ ss.

## 4. Discussion

There has been no large-scale experimental characterization of splicing efficiency, so it is difficult to relate PWMS as a proxy of splicing strength to splicing efficiency. However, the three yeast ICGs whose splicing depends on Mer1p have experimentally measured splicing efficiency expressed as percentage of transcripts spliced in the wild type [73]. When *Mer1* is not expressed, these percentages are 32% and 31% for *AMA1*, 14% and 13% for *REC107/Mer2*, and 3% and 4% for *HFM1/Mer3*. The corresponding values when *Mer1* is expressed are 71% and 72% for *AMA1*, 53% and 59% for *REC107/Mer2*, and 42% and 42% for *HFM1/Mer3*. Consistent with this ranking of splicing efficiency of *AMA1 *> *REC107/Mer2 *> *HFM1/Mer3, *5′ ss and 3′ ss PWMS values are 8.7838 and 11.4573 for *AMA1, *4.6995, and 5.6668 for *REC107/Mer2*, and −7.3825 and 1.3488 for *HFM1/Mer3. *Thus, in this limited case, the experimentally measured splicing efficiency shows excellent concordance with PWMS.

Three additional but indirect lines of evidence suggest that PWMS is an appropriate proxy for splicing strength. First, PWMS for both 5′ ss and 3′ ss is positively correlated with gene expression. Second, introns spliced by nonspliceosomal mechanisms or requiring additional protein factors for splicing generally have low PWMS. Third, yeast ICGs that recruit splicing factors poorly tend to have lower PWMS than those that bound well to splicing factors. These results suggest the potential of using PWMS as a screening tool for ICGs not spliced by the spliceosome mechanism or requiring additional regulatory factors for splicing in other fungal species.

The characterized 5′ ss (UA***AAG***∣***GUAUGUU***UAAUU, where significant sites are in bold italic) and 3′ ss (***UUUUUUUUAYAG***∣GCUUC) in the yeast expanded the conventional yeast consensus splice sites. While the +1 site in the 3′ ss site is not statistically significant after adjusting for experimentwise error rate, a recent experimental study suggests that it does affect splicing efficiency. The overused nucleotide at this site is G, followed by A, but C is strongly avoided (Table 4). Changing the +1A to +1C in the *LSM7* mRNA resulted in splicing at a downstream AG∣A site [81]. In *REC102 *gene, the splicing at the normal 3′ ss site UGAAG∣A site is reduced when the +1A is changed to C, especially when nucleotide C in an upstream AG∣C site is changed to A [81]. Our results (Table 4) showing the preference of +1R and avoidance of −1C corroborate these experimental studies and suggest that the preference of +1R and avoidance of −1C may be a general feature of splicing by yeast spliceosome.

Selection for increased splicing strength is expected to be stronger in highly transcribed genes than in lowly transcribed genes. Consistent with this expectation, splicing strength is higher for highly transcribed ICGs than for lowly transcribed ICGs and is higher for ICGs whose mRNAs are efficiently translated than those whose mRNAs are not efficiently translated. It has long been known that highly expressed yeast genes exhibit a high degree of codon-anticodon adaptation [91]. Our result here suggests that natural selection is also operating on the splicing machinery.

The presence of poly-U immediately before the 3′ AG in 3′ ss, instead of a polypyrimidine tract in other eukaryotes, may arise from the following evolutionary process. The polypyrimidine is recognized by, and bound to, U2AF^65^ in other eukaryotes including *S. pompe*. The U2AF^65^ homologue in the budding yeast, MUD2p, is highly diverged and not essential for survival [20]. This may have contributed to a weakened selection constraint on the evolution of the polypyrimidine tract in the budding yeast. Because the budding yeast genome is AT rich, nucleotide C may be progressively replaced by nucleotide T, leading to the transition of the polypyrimidine tract to the poly-U tract. However, this mutationist hypothesis cannot explain why the poly(U) can increase splicing efficiency. It is likely that both mutation and selection participated in the evolution of poly(U) in the yeast intron before the 3′ AG.

We should mention that splicing efficiency of yeast introns depends not only on 5′ ss and 3′ ss, but also on the branchpoint sequence (BPS, [92–94]) as well as the spacing between BPS and 3′ ss [95–97]. For example, both 5′ and 3′ ss of *Yra1 *are strong with high PWMS, but the intron splicing is regulated, possibly through its unconventional branchpoint site GACUAAC (in contrast to the consensus UACUAAC).

## Figures and Tables

**Table 1:** The names and intron positions of 24 yeast protein-coding genes which have introns in their 5′-UTRs.

Syst. name^(1)^	Std name^(2)^	Chr	Position^(3)^	Genome position	Strand^(4)^
YBL072C	RPS8A	2	-315..-8	89440..89133	C
YBL092W	RPL32	2	-333..-1	45645..45977	W
YBR089C-A	NHP6B	2	-384..-28	426873..426517	C
YDL061C	RPS29B	4	-421..-13	341219..340811	C
YDL137W	ARF2	4	-371..-40	216158..216489	W
YDL189W	RBS1	4	-138..-40	122078..122176	W
YDR099W	BMH2	4	-826..-84	652781..653523	W
YER102W	RPS8B	5	-367..-8	362733..363092	W
YER131W	RPS26B	5	-361..-1	423591..423951	W
YFR032C-A	RPL29	6	-334..-4	223771..223441	C
YGL031C	RPL24A	7	-463..-8	438397..437942	C
YGL187C	COX4	7	-354..-13	150525..150184	C
YGL189C	RPS26A	7	-378..-11	148966..148599	C
YGR027C	RPS25A	7	-327..-16	534785..534474	C
YGR148C	RPL24B	7	-399..-8	788178..787787	C
YIL123W	SIM1	9	-489..-3	127662..128148	W
YJL130C	URA2	10	-385..-66	172752..172433	C
YKL150W	MCR1	11	-144..-57	166400..166487	W
YKL186C	MTR2	11	-167..-14	93465..93312	C
YLR333C	RPS25B	12	-436..-14	796335..795913	C
YLR367W	RPS22B	12	-564..-8	855878..856434	W
YLR388W	RPS29A	12	-493..-6	898158..898645	W
YNL066W	SUN4	14	-358..-13	501157..501502	W
YPL230W	USV1	16	-93..-19	115219..115293	W

^(1)^Systematic name.

^(2)^Standard name.

^(3)^Site numbering relative to start codon.

^(4)^C—Crick strand (reverse complement), W—Watson strand.

**Table 2:** Yeast genes whose first exon (i.e., the coding part of the first exon) is shorter than five nucleotides.

Gene	PWM*	1st Exon len	Sequence
BET4	4.2977	3	AUG
BOS1	3.5760	3	AUG
DCN1	6.5363	3	AUG
MND1	8.1685	3	AUG
MPT5	8.5055	3	AUG
PSP2	8.4546	4	AUGG
QCR9	5.7592	3	AUG
RPL13A	6.9991	4	AUGG
RPL13B	8.6752	4	AUGG
RPL19A	6.9298	2	AU
RPL19B	11.7762	2	AU
RPL20A	9.7145	1	A
RPL20B	8.0214	1	A
RPL2A	12.0769	4	AUGG
RPL2B	9.7326	4	AUGG
RPL30	8.1799	3	AUG
RPL35A	7.3834	3	AUG
RPL35B	7.8326	3	AUG
RPL42A	9.2392	4	AUGG
RPL42B	7.0558	4	AUGG
RPL43A	9.9976	2	AU
RPL43B	12.0547	2	AU
RPS17A	9.1269	3	AUG
RPS17B	10.1283	3	AUG
RPS24A	9.4227	3	AUG
RPS24B	11.3548	3	AUG
RPS27A	6.3612	3	AUG
RPS27B	10.3823	3	AUG
RPS30A	10.8845	3	AUG
RPS30B	6.2290	3	AUG
UBC12	8.4505	3	AUG
VMA10	8.2722	3	AUG
YSF3	7.1596	3	AUG

*Position weight matrix score at 3′ ss.

**Table 3:** Site-specific frequencies and position weight matrix (PWM) for 275 5′ ss. The consensus sequence (UA***AAG ***∣***GUAUGUU ***UAAUU) can be obtained from those large site-specific PWM entries, with the most important sites in ***bold italics ***. The *χ* ^2^ test is performed for each site against the background frequencies (A = 0.3279, C = 0.1915, G = 0.2043, and U = 0.2763). The nucleotide sites are labeled with the five exon nucleotides as −5 to −1 and the 12 intron nucleotides as 1 to 12. The PWM is nearly identical when the introns in 5′ UTR were excluded.

Site	A	C	G	U	*χ* ^2^	*P*	A	C	G	U
−5	94	32	57	92	11.798	0.0081088	0.0641	−0.7117	0.0245	0.2792
−4	119	47	48	61	14.117	0.0027505	0.4032	−0.1599	−0.2225	−0.3115
−3	139	38	43	55	39.672	0.0000001	***0.6268***	−0.4651	−0.3805	−0.4601
−2	138	40	36	61	38.899	0.0000001	***0.6164***	−0.3915	−0.6355	−0.3115
−1	91	45	88	51	27.270	0.0000052	0.0174	−0.2223	***0.6492***	−0.5685
1	0	1	274	0	1060.426	0.0000004	−8.1042	−5.4675	***2.2855***	−8.1044
2	0	9	0	266	658.096	0.0000003	−8.1042	−2.5200	−8.1048	***1.8081***
3	268	1	2	4	522.754	0.0000003	***1.5723***	−5.4675	−4.6732	−4.1523
4	17	29	1	228	428.607	0.0000002	−2.3805	−0.8528	−5.5454	***1.5859***
5	2	0	272	1	1041.047	0.0000004	−5.2765	−8.1049	***2.2750***	−5.8967
6	10	8	2	255	583.545	0.0000003	−3.1271	−2.6862	−4.6732	***1.7472***
7	97	18	39	121	55.570	0.0000001	0.1092	−1.5351	−0.5206	***0.6734***
8	95	54	35	91	11.363	0.0099180	0.0793	0.0397	−0.6759	0.2635
9	123	45	34	73	22.172	0.0000601	0.4508	−0.2223	−0.7175	−0.0534
10	118	41	38	78	17.334	0.0006034	0.3911	−0.3560	−0.5579	0.0418
11	105	33	43	94	17.367	0.0005940	0.2232	−0.6676	−0.3805	0.3101
12	90	44	42	99	12.109	0.0070180	0.0015	−0.2546	−0.4142	0.3847

**Table 4:** Site-specific frequencies and position weight matrix (PWM) for 301 3′ ss. The consensus sequence (***UUUUUUUUAYAG ***∣GCUUC) can be obtained from those large site-specific PWM entries, with the most important sites in ***bold italics ***. The *χ* ^2^ test is performed for each site against the expected background frequencies. The sites are labeled with first-exon site as 1. The PWM is nearly identical when the introns in 5′ UTR were excluded.

Site	A	C	G	U	*χ* ^2^	*P*	A	C	G	U
−12	70	58	37	136	51.729	0.0000001	−0.4898	0.0122	−0.7264	***0.7114***
−11	79	51	23	148	79.511	0.0000001	−0.3161	−0.1727	−1.4074	***0.8332***
−10	86	45	14	156	105.131	0.0000001	−0.1941	−0.3525	−2.1155	***0.9090***
−9	43	33	23	202	236.063	0.0000001	−1.1886	−0.7978	−1.4074	***1.2812***
−8	56	43	31	171	130.216	0.0000001	−0.8100	−0.4178	−0.9801	***1.0412***
−7	102	35	31	133	54.256	0.0000001	0.0512	−0.7134	−0.9801	***0.6793***
−6	103	46	38	114	23.130	0.0000380	0.0653	−0.3210	−0.6881	***0.4574***
−5	100	36	25	140	68.925	0.0000000	0.0228	−0.6729	−1.2882	***0.7532***
−4	145	27	41	88	45.473	0.0000001	***0.5574***	−1.0854	−0.5790	0.0850
−3	15	127	0	159	284.824	0.0000002	−2.6877	***1.1404***	−8.2350	***0.9364***
−2	299	1	1	0	605.789	0.0000003	***1.5998***	−5.5977	−5.6756	−8.2346
−1	0	0	301	0	1171.443	0.0000004	−8.2345	−8.2351	***2.2908***	−8.2346
1	109	39	74	79	9.936	0.0191208	0.1467	−0.5580	0.2697	−0.0701
2	84	66	55	96	6.036	0.1098600	−0.2279	0.1981	−0.1571	0.2102
3	103	58	50	90	2.969	0.3964877	0.0653	0.0122	−0.2940	0.1173
4	96	45	56	104	8.655	0.0342400	−0.0359	−0.3525	−0.1312	0.3253
5	100	69	39	93	11.698	0.0084938	0.0228	0.2620	−0.6508	0.1645

**Table 5:** Evaluating statistical significance of individual nucleotide sites (site, with 5 nucleotides on the exon side labelled −5 to −1 and 12 on the intron side labeled 1 to 12) of 5′ ss by two types of false discovery rate.

Site	*P*	pBH^(1)^	pBY^(2)^
1	*0.0000000000^†^	0.002941	0.000855
5	*0.0000000000^†^	0.005882	0.001710
2	*0.0000000000^†^	0.008824	0.002565
6	*0.0000000000^†^	0.011765	0.003420
3	*0.0000000000^†^	0.014706	0.004276
4	*0.0000000000^†^	0.017647	0.005131
7	*0.0000000000^†^	0.020588	0.005986
−2	*0.0000004842^†^	0.023529	0.006841
−3	*0.0000013734^†^	0.026471	0.007696
−1	*0.0000030965^†^	0.029412	0.008551
9	*0.0002619304^†^	0.032353	0.009406
10	*0.0006307900^†^	0.035294	0.010261
12	*0.0025004071^†^	0.038235	0.011116
11	*0.0033589734^†^	0.041176	0.011971
8	*0.0084455695^†^	0.044118	0.012827
−5	*0.0177349476	0.047059	0.013682
−4	*0.0182291629	0.050000	0.014537

^(1)^Critical *P* based on [54].

^(2)^Critical *P* based on [55].

*Significant by the criterion in [54].

^†^Significant by the criterion in [55].

**Table 6:** Position weight matrix scores (PWMSs, as a proxy for splicing strength) is significantly smaller for splice sites from intron-containing genes (ICGs) whose transcripts failed to recruit U1 snRNPs (NRG for nonrecruiting group) than for those from ICGs whose transcripts bind well to U1 snRNPs (RG for recruiting group). The pattern is consistent for both 5′ ss and 3′ ss, based on two-sample *t*-tests assuming equal variances. Mann-Whitney tests yield the same conclusion.

	5′ ss		3′ ss	
	NRG	RG	NRG	RG
PWMS mean	8.8138	11.1978	5.3129	7.1762
PWMS Var.	31.5069	4.8646	13.3017	8.2077
*N*	44	231	49	252
*t*	−4.6346		−3.9257	
*P*	0.0000		0.0001	

**Table 7:** Testing the predictions that introns in highly expressed genes have higher PWMS and smaller variance in PWMS than in lowly expressed genes, with gene expression measured by CAI, mRNA, and protein abundance. Introns spliced by nonspliceosomal mechanisms are excluded. Mann-Whitney tests generate similar results. All tests are two tailed. The results are nearly identical when mRNA abundance from microarray [27] is used instead of that from GATC-PCR [28] or when protein abundance [29] is used instead of the protein synthesis rate [30].

	5′ ss			3′ ss		
	CAI	lnMRNA^(1)^	lnPROT^(2)^	CAI	lnMRNA	lnPROT
N^(3)^	91	48	55	100	53	67
MeanH^(4)^	11.4927	11.3128	11.4879	8.7188	8.5447	8.6004
MeanL^(5)^	9.4135	9.7143	9.7581	5.1109	4.9729	5.3359
DF^(6)^	113	59	55	155	83	82
*T*	4.1635	2.2501	2.2411	9.7833	6.9719	5.7687
*P*	0.0001	0.0282	0.0291	<0.0001	<0.0001	<0.0001

VarH^(7)^	3.2053	2.8657	2.7345	2.6326	3.4395	3.4220
VarL^(8)^	10.3934	21.3602	24.6692	20.0609	10.4712	9.9223
*F*	3.2429	7.4537	9.0214	7.6211	3.0444	2.9000
*P*	<0.0001	<0.0001	<0.0001	<0.0001	<0.0001	0.0001

^(1)^Natural logarithm of mRNA abundance [28].

^(2)^Natural logarithm of protein synthesis rate [30].

^(3)^Number of ss in the highly expressed and lowly expressed groups (note that *N*_1_ = *N*_2_ = *N*).

^(4)^Mean PWMS in highly expressed group.

^(5)^Mean PWMS in lowly expressed group.

^(6)^The *t*-test assuming unequal variance is used. SoDF is not equal to (*N*_1_ + *N*_2_ − 2).

^(7)^Variance in the highly expressed group.

^(8)^Variance in the lowly expressed group.
